# The evolutionary history of the catenin gene family during metazoan evolution

**DOI:** 10.1186/1471-2148-11-198

**Published:** 2011-07-08

**Authors:** Zi-Ming Zhao, Albert B Reynolds, Eric A Gaucher

**Affiliations:** 1 School of Biology, Georgia Institute of Technology, Atlanta, GA 30332, USA; 2 Department of Cancer Biology, Vanderbilt University, Nashville, TN, 37232, USA

## Abstract

**Background:**

Catenin is a gene family composed of three subfamilies; p120, beta and alpha. Beta and p120 are homologous subfamilies based on sequence and structural comparisons, and are members of the armadillo repeat protein superfamily. Alpha does not appear to be homologous to either beta or p120 based on the lack of sequence and structural similarity, and the alpha subfamily belongs to the vinculin superfamily. Catenins link the transmembrane protein cadherin to the cytoskeleton and thus function in cell-cell adhesion. To date, only the beta subfamily has been evolutionarily analyzed and experimentally studied for its functions in signaling pathways, development and human diseases such as cancer. We present a detailed evolutionary study of the whole catenin family to provide a better understanding of how this family has evolved in metazoans, and by extension, the evolution of cell-cell adhesion.

**Results:**

All three catenin subfamilies have been detected in metazoans used in the present study by searching public databases and applying species-specific BLAST searches. Two monophyletic clades are formed between beta and p120 subfamilies using Bayesian phylogenetic inference. Phylogenetic analyses also reveal an array of duplication events throughout metazoan history. Furthermore, numerous annotation issues for the catenin family have been detected by our computational analyses.

**Conclusions:**

Delta2/ARVCF catenin in the p120 subfamily, beta catenin in the beta subfamily, and alpha2 catenin in the alpha subfamily are present in all metazoans analyzed. This implies that the last common ancestor of metazoans had these three catenin subfamilies. However, not all members within each subfamily were detected in all metazoan species. Each subfamily has undergone duplications at different levels (species-specific, subphylum-specific or phylum-specific) and to different extents (in the case of the number of homologs). Extensive annotation problems have been resolved in each of the three catenin subfamilies. This resolution provides a more coherent description of catenin evolution.

## Background

Catenin (derived from "catena", "chain" in Latin) is a gene family that links the transmembrane protein cadherin to the cytoskeleton and functions in cell-cell adhesion [1]. The catenin family is composed of three subfamilies; p120 subfamily, beta subfamily and alpha subfamily. The p120 subfamily includes seven members, which are p120 (also named delta1 catenin), Armadillo Repeat protein deleted in Velo-Cardio-Facial syndrome (ARVCF), delta2 catenin (also named NPRAP/Neurojungin), plakophilin (pkp) 4 (also named p0071), pkp1, pkp2, and pkp3 [2]. The beta subfamily includes gamma catenin (also named plakoglobin) in addition to beta catenin [3]. The alpha subfamily includes alpha1 catenin (also named alpha-E-catenin), alpha2 catenin (also named alpha-N-catenin) and alpha3 catenin (also named alpha-T-catenin) [4].

The biomolecular functions and evolutionary history for the catenin family have not been well studied except for beta catenin [3]. Beta catenin functions in both cadherin-associated cell adhesion (adherens junction) and Wnt signaling pathways. This subfamily participates in development and human diseases such as cancer [3,5]. Previous evolutionary analyses demonstrate that gamma catenin duplicated from beta catenin in vertebrates and there have been two separate beta catenin duplications specific to *Caenorhabditis elegans *[3]. Gamma (also named plakoglobin) and the four plakophilins are all components of the desmosome [6]. Previous studies have also shown a relationship between p120 and human cancer [2], while limited evolutionary information has been revealed for the p120 subfamily [7]. The detailed functions and histories of the p120 and alpha subfamilies, however, have not yet been extensively studied. Thus evolutionary studies for the whole catenin family seem appropriate given the sufficient amount of metazoan sequence data currently available and the interest of the biomedical community in the catenin family. Additionally, the availability of the genomic sequence from the premetazoan unicellular choanoflagellate *Monosiga brevicollis *[8] enables us to make inferences about the catenin family before the emergence of metazoans.

In the present study, we analyzed the gene presence/absence for members of the catenin family from fully sequenced representative metazoans by searching public databases and applying species-specific BLAST searches. All three subfamilies (but not all members of each subfamily) are present in all metazoan species analyzed here. This implies that the last common ancestor of metazoans had all three catenin subfamilies, but members of catenin subfamilies have duplicated at different points in evolutionary history. We applied Bayesian phylogenetic analysis for the p120 and beta subfamilies together, and each of the three catenin subfamilies separately. The results demonstrate that p120 and beta catenins form monophyletic groups and that catenins have undergone multiple duplications at different levels (species-specific, subphylum-specific or phylum-specific) and to different extents (in the case of the number of paralogs).

Our bioinformatics and evolutionary analyses have also helped to resolve some existing annotation issues associated with catenin members in both vertebrates and non-vertebrates [9]. The annotation issues include wrong annotations, confusing annotations (different names related or not related to the true name), and hypothetical annotations. Confusing or hypothetical annotations for unknown genes or gene families exist because they have not been functionally well characterized. Wrong annotations occur frequently in highly similar or highly divergent homologs, since it is hard to resolve annotation by sequence information alone [9]. Thus, the application of evolutionary analyses to the annotation of new sequences in large gene families can hold considerable value.

## Results

### Origins of the catenin subfamily members during metazoan evolution

**Figure 1:**
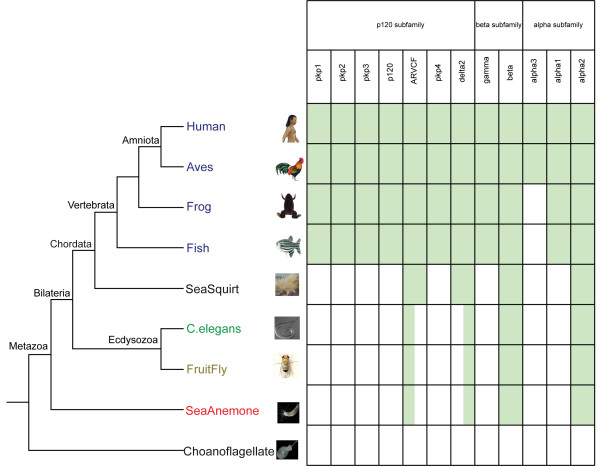
**The presence/absence of catenin family members in species during metazoan evolution**. The cladogram of metazoans on the left is based on metazoan evolution with the premetazoan choanoflagellate as the outgroup. Species have been selected in the phyla of Vertebrata, Urochordata, Arthropoda, Nematoda, and Cnidaria to represent metazoans. Vertebrata and Urochordata together formed the Chordata phylum, Arthropoda and Nematoda formed the Ecdysozoa clade, Chordata and Ecdysozoa formed the Bilateria clade, and Bilateria and Cnidaria containing Radiata formed the Metazoa clade. We applied different colors for species in different phyla, such as blue for all vertebrates, black for the urochordate sea squirt and the premetazoan choanoflagellate, green for the nematode *C. elegans*, brown for the arthropod fruit fly, and red for the cnidarian sea anemone. Same color patterns were applied for species from different phyla in Figure 3, Figure 4, Additional file 1, Additional file 2 and Additional file 3. Based on the species tree, we determined presence/absence of catenin family members. The table on the right side of the species tree includes all catenin genes, and each shaded green box indicates the presence of the specific gene in the particular species shown in the tree, while the white box indicates the absence of the specific gene. The partially-shaded green boxes indicate unresolved presence/absence of the delta2 and ARVCF members. These species had either a delta2 or an ARVCF but the current analyses cannot resolve the relationship.

Figure 1 shows a cladogram for metazoan evolution [10] with the premetazoan choanoflagellate *M. brevicollis *as the outgroup used for our study. We selected species in the phyla of Vertebrata, Urochordata, Arthropoda, Nematoda, and Cnidaria to represent metazoans. We used Mammalia, Aves, Amphibia and Ray-finned fish to represent classes for Vertebrata; *Homo sapiens *(human) for Mammalia, *Gallus gallus *(chicken) or *Taeniopygia guttata *(finch) for Aves, *Xenopus laevis *or *Xenopus tropicalis *(frog) for Amphibia, and *Danio rerio *(fish) for Ray-finned fish. For Aves and Amphibia, chicken/finch and *X. laevis/X. tropicalis *were used depending on the sequence availability, sequence length and quality. We used *Ciona intestinalis *(sea squirt) for Urochordata, *Drosophila *(fruit fly) and/or other insects for Arthropoda, *C. elegans *and/or others for Nematoda, *Nematostella vectensis *(sea anemone) and/or *Hydra magnipapillata *for Cnidaria. Vertebrata and Urochordata together formed the Chordata phylum, and Bilateria and Cnidaria containing Radiata formed the Metazoa clade. Based on this species tree of metazoan evolution, we determined presence/absence of catenin family members.

In Figure 1, green shaded boxes in the table indicate the presence of catenin family members detected in the representative organisms from the species tree. The distribution demonstrates that all three subfamilies have been detected in all the selected metazoan species but not in the premetazoan *M. brevicollis*. Furthermore, we performed presence/absence analyses to other non-metazoans including the amoeba *Dictyostelium discoideum*, the protist *Cryptosporidium parvum*, three fungi (*Aspergillus, Saccharomyces*, and *Schizosaccharomycetes*), three unicellular algae (*Ostreococcus, Chlamydomonas*, and *Thalassiosira*), and the plant *Arabidopsis*. None of the catenin members have been detected in the above non-metazoans. We infer that these three catenin subfamilies were therefore present in the last common ancestor of metazoans, but none were present in non-metazoans. All members of the p120 subfamily except ARVCF and delta2 are present in only vertebrates. This scenario also holds for gamma catenin from the beta subfamily and alpha1 catenin from the alpha subfamily. The annotation of the catenin members in non-vertebrates is based on extensive analyses and discussed in the annotation section.

We further extended presence/absence analyses to other non-catenin gene families involved in desmosome formation. These included both desmosomal cadherins desmogleins and desmocollins, as well as desmoplakins. All of these genes have been detected only in vertebrates but not detected in non-vertebrates.

### Evolution of an ancient duplication in the catenin family

**Figure 2:**
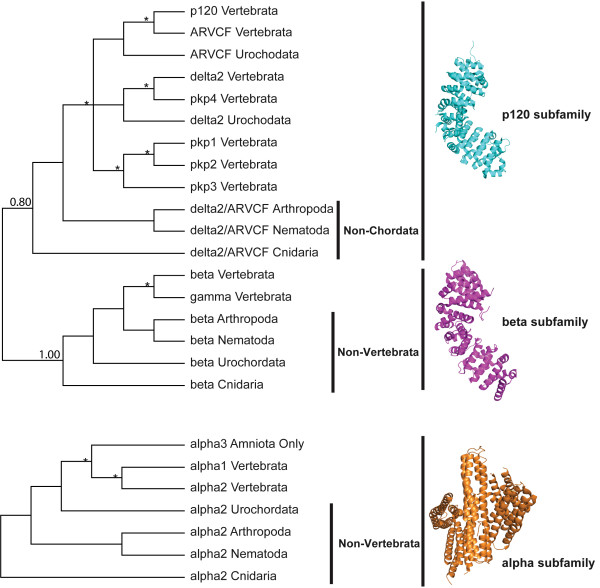
**The evolution of the catenin family**. The cladogram of each catenin subfamily is based on the individual subfamily topology with sea anemone from the Cnidaria phylum as the outgroup. The tree was condensed to the higher level (the phylum level), including the phyla of Vertebrata, Urochordata, Arthropoda, Nematoda, and Cnidaria. Non-Chordata and Non-Vertebrata were labelled additionally. The taxon names in the tree are a combination of the gene name and the phylum name. The p120 subfamily and the beta subfamily are homologous, thus were shown together in the phylogeny. Asterisks indicate duplication events. The tertiary structures were presented along the phylogeny, with PDB ID accessions 3L6X, 2Z6G, and 1L7C for the p120, beta and alpha subfamilies, respectively.

Figure 2 shows that the p120 subfamily forms a monophyletic clade supported by a posterior probability (PP) equal to 0.8 and the beta subfamily forms a separate monophyletic clade (PP = 1.0). The alpha subfamily does not appear to be homologous with p120/beta according to sequence and structural analyses. Representative tertiary structures from the three catenin subfamilies were extracted from the Protein Data Bank [11]: PDB ID accessions 3L6X, 2Z6G, and 1L7C for the p120, beta and alpha subfamilies, respectively. Figure 2 shows cartoon representations of the tertiary structures for the alpha and the p120/beta subfamilies. To the eye, these structures clearly lack analogous folds. Specifically, the p120/beta subfamilies contain armadillo domain (ARM) repeats, while the alpha subfamily contains vinculin homolog domains. An attempt to align the three subfamilies failed to identity conserved anchors that would have allowed us to align homologous regions. Thus, our structural and sequence analyses suggest the alpha subfamily is not evolutionarily related to the p120/beta subfamilies, and the catenin family has undergone an ancient duplication resulting in the p120 and beta subfamilies.

### Origins and duplications of p120 subfamily members

**Figure 3:**
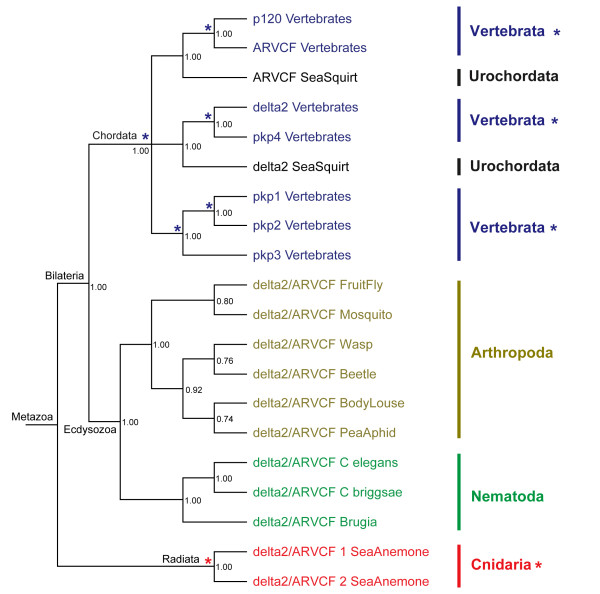
**The evolution of the p120 subfamily**. The Bayesian phylogeny is supported with posterior probabilities, having sea anemone as the outgroup. Asterisks indicate duplication events. Coloring scheme is identical to Figure 1.

Figure 3 shows the Bayesian phylogeny for p120 subfamily members with delta2/ARVCF catenin from sea anemone serving as the outgroup. The complete tree with branch lengths and NCBI sequence identifiers is shown in Additional file 1. Figure 3 shows that p120 and ARVCF in vertebrates form a clade (PP = 1.0) with ARVCF from sea squirt as the outgroup (PP = 1.0). This figure also shows that delta2 catenin and pkp4 in vertebrates form a clade (PP = 1.0) with delta2 from sea squirt as the outgroup (PP = 1.0). Figure 3 shows that pkp1, pkp2 and pkp3 in vertebrates form a clade (PP = 1.0) within which pkp1 and pkp2 form a monophyletic clade (PP = 1.0). All of the above genes in chordates form a monophyletic clade (PP = 1.0) with all non-chordate delta2/ARVCF positioned outside the clade. In total, these results suggest that: p120 and ARVCF in vertebrates share a common ancestor with ARVCF in the urochordate; delta2 and pkp4 in vertebrates share a common ancestor with delta2 catenin in the urochordate; pkp1, pkp2 and pkp3 in vertebrates share a common ancestor, and all of the above genes share a common ancestor with delta2/ARVCF catenin in non-chordates.

Our analyses suggest that the seven members of the p120 subfamily in vertebrates emerged by non-contemporaneous duplication events but the majority of these subfamily members (five out of seven) occurred with the origin of vertebrates. Delta2 and ARVCF diverged with the origin of chordates while p120 duplicated from ARVCF with the origin of vertebrates and pkp4 duplicated from delta2 catenin with the origin of vertebrates. It is unclear exactly which member of the p120 subfamily pkp1, pkp2 and pkp3 duplicated from since this monophyletic group does not confidently clade with either ARVCF or delta2 from the urochordate. We can assume, however, that pkp1, pkp2 and pkp3 ultimately evolved from delta2/ARVCF because the ancestor of chordates appears to have had only one p120 member (delta2/ARVCF).

In addition to vertebrate-specific subphylum and chordate-specific phylum duplications, the p120 subfamily may have undergone species-specific duplications in Cnidaria. We cannot rule out the possibility that this duplication occurred at a higher level since only a single cnidarian species was incorporated in our analyses. Support of the former scenario, however, comes from an analysis of two truncated p120 subfamily homologs from *Hydra *(with NCBI protein identifiers 221130487 and 221131941 separately). A phylogenetic analysis groups the two sea anemone delta2/ARVCF as a monophyletic clade to the exclusion of the two delta2/ARVCF from *Hydra *that also form a monophyletic clade (not shown since the sequences are not complete). It is possible that delta2/ARVCF has undergone species-specific duplications in sea anemone and *Hydra *separately, but a more confident conclusion will depend on complete sequences from *Hydra *and/or the availability of sequences from other cnidarians.

In total, all p120 subfamily members share a common ancestor with delta2/ARVCF from non-chordates. This subfamily experienced multiple vertebrate-specific subphylum duplications, a single chordate-specific phylum duplication and possible species-specific duplications in the Cnidaria phylum.

### Origins and duplications of beta subfamily members

**Figure 4:**
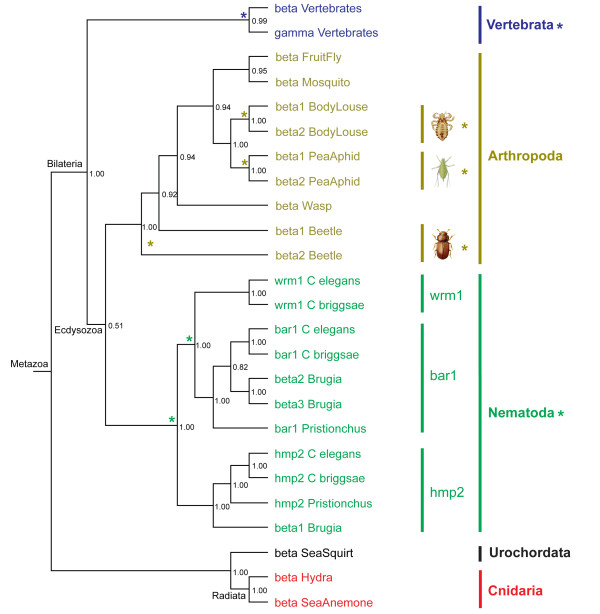
**The evolution of the beta catenin subfamily**. The Bayesian phylogeny is supported with posterior probabilities, having sea anemone as the outgroup. Asterisks indicate duplication events. Coloring scheme is identical to Figure 1.

Figure 4 shows the Bayesian phylogeny for beta subfamily members with beta catenin from sea anemone serving as the outgroup. The complete tree with branch lengths and NCBI sequence identifiers is shown in Additional file 2. Figure 4 shows that gamma and beta in vertebrates form a monophyletic clade (PP = 0.99) with all non-vertebrate betas positioned outside the clade. This result suggests that gamma and beta catenin in vertebrates share a common ancestor with beta in non-vertebrates, and gamma duplicated from beta with the origin of vertebrates.

In addition to a vertebrate-specific subphylum duplication, the beta subfamily has undergone species-specific and phylum-specific duplications. Figure 4 shows that in the Nematoda phylum, three monophyletic clades are formed by bar1, wrm1, and hmp2 separately (all having PP = 1.0), suggesting two Nematoda-specific phylum duplications. However, only two of the four species (*C. elegans *and *C. Briggsae*) studied here contain the complete three beta catenin paralogs, while *Pristionchus *and *Brugia *seem to have lost wrm1. Additionally, *Brugia *contains two beta paralogs (labelled as beta2 and beta3 arbitrarily by us) within the bar1 clade, indicating a *Brugia *species-specific duplication within the Nematoda phylum. Thus the beta subfamily has undergone a species-specific duplication in *Brugia *and two phylum-specific duplications.

For the Arthropoda phylum, two monophyletic clades (both having PP = 1.0) are formed from two individual species-specific duplications in the pea aphid and the body louse. Each group is the result of a single duplication resulting in two paralogs. Beetle also contains what appears to be a species-specific duplication but the paralogs do not group together so we cannot rule out an ancient origin for this duplication. As for the Cnidaria phylum, we have found two beta catenin homologs in sea anemone and only one in *Hydra*. We only show one beta catenin (442 amino acids, aa) for sea anemone in Figure 4 since the other potential paralog (with the NCBI protein identifier 156615300) has been truncated to 298 aa, and this short length makes it hard to resolve the phylogenetic position for this paralog. The potential presence of two beta catenin homologs in sea anemone indicates a possible species-specific duplication in the Cnidaria phylum.

In total, all beta subfamily members share a common ancestor with beta catenin from non-vertebrates. This subfamily experienced a vertebrate-specific subphylum duplication, two nematode-specific phylum duplications, a species-specific duplication in the Nematoda phylum, multiple species-specific duplications in the Arthropoda phylum and a possible species-specific duplication in the Cnidaria phylum. The conflict between the gene tree of the beta subfamily and the species tree of metazoan evolution for the position of the urochordate sea squirt is caused by a combination of slow evolution outside the nematode clade and rapid evolution inside the nematode clade (Additional file 2). This difference in evolutionary rates is supported by Wilcoxon rank and Kolmogorov-Smirnov tests, both with P values < 0.0001.

### Origins and duplications of alpha subfamily members

The alpha subfamily has not experienced extensive duplication events similar to the p120 and beta subfamilies. Thus, the evolutionary history of this gene subfamily is easier to resolve. Additional file 3 shows that alpha1 and alpha2 in vertebrates form two monophyletic clades (both having PP = 1.0) and together form a clade (PP = 1.0) to the exclusion of alpha3 - forming its own monophyletic clade (PP = 1.0). The two vertebrate-specific and one amniote-specific alphas form a monophyletic clade with all non-vertebrate alphas positioned outside the clade. Our analyses suggest alpha1 duplicated from alpha2 with the origin of vertebrates. It is unclear exactly which member of the alpha subfamily alpha3 duplicated from since this monophyletic group does not confidently clade with either alpha1 or alpha2 from vertebrates. We can assume, however, that alpha3 ultimately evolved from alpha2 because the ancestor of vertebrates appears to have had only one alpha member (alpha2). Our results show that the alpha subfamily experienced a vertebrate-specific subphylum duplication and an amniote-specific duplication. In total, all alpha subfamily members share a common ancestor with alpha2 from non-vertebrates.

### Annotation issues for the catenin family

Our evolutionary studies have resolved annotation issues in all three catenin subfamilies (Additional file 4) and have allowed us to identify nomenclature of catenin genes present before the diversification of vertebrates. Some issues are similar between subfamilies while other issues are unique to specific subfamilies.

The urochordate sea squirt contains a p120 subfamily homolog that has been annotated as 'p120'. Our analysis shows that the sea squirt sequence annotated as p120 is slightly more similar in sequence identity to ARVCF members in vertebrates (36-41%) than p120 members in vertebrates (36-40%). We therefore had to perform additional analyses to resolve this ambiguity. First, a BLASTP search using this sea squirt 'p120/ARVCF' as the query against only p120s and ARVCFs from vertebrates was carried out. This analysis demonstrated that all ARVCFs have higher BLASTP scores (bits) and lower E-values to the sea squirt sequence than the p120s from vertebrates. Second, we performed MEGA pairwise distance comparisons of the sea squirt 'p120/ARVCF' to vertebrate p120s and ARVCFs using both Dayhoff and JTT models (no. of sites = 728aa). Both models gave the same results; the distances between the sea squirt 'p120/ARVCF' and the vertebrate ARVCFs are shorter than the distances between the sea squirt 'p120/ARVCF' and the vertebrate p120s, and this difference in distance is statistically significant (P value equals 0.05) if both human ARVCF and p120 are removed from the analysis. We extended these analyses to include measures of functional divergence. We used the DIVERGE software package to identify type-II functional sites (a site that is conserved-but-different between clades) to help resolve the annotation of the sea squirt 'p120/ARVCF'. Of the 70 type-II sites identified by DIVERGE, 36% of sites (25) support the annotation of sea squirt as ARVCF while only 20% (14) support the annotation as p120. The remaining sites do not have resolution in favor of one annotation over the other. Thus, the BLASTP, MEGA and DIVERGE analyses all suggest that the sea squirt 'p120/ARVCF' is more similar to the ARVCF group than the p120 group.

The other p120 subfamily homolog in the urochordate sea squirt has been annotated as 'delta2'. Given the close relationship between delta2 and pkp4 (Figure 3 and Additional file 1), we applied the same analyses used for the sea squirt 'p120/ARVCF' to resolve the annotation of the sea squirt 'delta2/pkp4'. Both BLASTP and MEGA analyses suggest that the sea squirt 'delta2/pkp4' is closer to delta2 than pkp4. The MEGA result is statistically significant as supported by Wilcoxon rank and Kolmogorov-Smirnov tests, both with P values equal to 0.01. DIVERGE analysis further supported the annotation of the sea squirt sequence as delta2. Of the 88 type-II sites identified by DIVERGE, 32% of sites (28) support the annotation of sea squirt as delta2 while only 17% (15) support the annotation as pkp4. The remaining sites do not have resolution in favor of one annotation over the other. This implies that delta2 members evolved prior to the evolution of pkp4 members. Accordingly, we retain the original annotation of this sea squirt gene as delta2.

In regards to the p120 subfamily, only two p120 subfamily members are present in the urochordate sea squirt. This indicates they are the two oldest members of this subfamily. Bayesian phylogenetic analysis confirmed that all the p120 subfamily homologs in non-chordates share a common ancestor with all seven members of the p120 subfamily in vertebrates (Additional file 1). This phylogeny, however, does not provide conclusive evidence for which of the two oldest p120 subfamily members (ARVCF or delta2) gave rise to the vertebrate expansion of the subfamily. Therefore we performed extensive BLASTP analyses and series of MEGA pairwise distance comparisons to infer whether delta2 or ARVCF may be the more ancient member of the p120 subfamily. Both BLASTP and MEGA analyses suggest that the p120 subfamily homologs in non-chordates are closer to delta2 than ARVCF. This conclusion holds for all vertebrates except when the urochordate sea squirt is included in the analysis, and this is probably caused by rapid evolution as seen from the long branches leading to sea squirt delta2 and sea squirt ARVCF (Additional file 1). The MEGA analysis, however, is not statistically significant. Further, a DIVERGE analysis did not reveal sites that could help resolve the annotation of non-chordate p120 subfamily members. As such, it is not possible to apply one particular annotation to the non-chordates over another annotation.

Our analyses point to a delta2/ARVCF origin for the entire p120 subfamily since only delta2 or ARVCF is inferred to exist in non-chordates and the diversification of this subfamily occurred with the evolution of chordates. Unfortunately, gene annotation does not follow this pattern. Numerous non-chordate homologs in the p120 subfamily have been annotated as p120, armadillo repeat protein, plakophilins or fibronectins. To resolve these discrepancies we performed three BLASTP analyses using the 'mis-annotated' homologs as queries; #1) a BLASTP search of the non-redundant protein database, #2) a BLASTP search of the human protein database, and #3) a BLASTP search of the individual genomes from which the mis-annotated homolog resides. For both analyses #1 and #2, all queries resulted in trusted top-hits annotated as p120 subfamily members. For analysis #3, only a single homolog was identified in the individual genomes of arthropods and nematodes while searches of the cnidarian genomes retrieved two homologs (as discussed above, this duplication appears to be species-specific). We, therefore, annotated all these p120 subfamily homologs in non-chordates as delta2/ARVCF (Additional file 4).

For the beta subfamily, our presence/absence and phylogenetic analyses suggest that gamma catenin is the result of a duplication event from beta catenins that took place during the origin of vertebrates and thus there should not be any gamma catenins present in non-vertebrates. One gene from *S. mansoni*, however, is annotated as gamma catenin. We performed the same series of three BLAST searches described above. These analyses suggest that this homolog is neither more similar in sequence to gamma nor beta as it appears to have experienced an episode of rapid evolution along the branch leading to *S. mansoni *(data not shown). However, since our presence/absence and phylogenetic analyses suggest that only beta is present in non-vertebrates, we infer that this gene evolved from a beta catenin and has since lost most of the sequence signatures that separate beta from gamma (it has retained enough signatures that allow us to confidently identify this as a member of the beta subfamily). Thus our analyses suggest that this gene arose from a duplication event of beta catenin in *S. Mansoni *and we re-annotated this gamma as beta catenin (Additional file 4). *Aplysia californica *is the other species from the same phylum as *S. mansoni *that has whole genome (shotgun) sequence available. A BLAST search of this genome reveals only a single gene belonging to the beta subfamily. This suggests that the paralogs in *S. mansoni *arose via a species-specific duplication event. In total, our analyses suggest that beta is the most ancient member of this subfamily with gamma arising during the origin of vertebrates while other duplication events have taken place in individual species such as *S. mansoni*.

For the alpha subfamily, our presence/absence and phylogenetic analyses suggest a vertebrate origin for alpha1 catenin. Both BLASTP and MEGA analyses suggest the alpha subfamily member present in non-chordates is more similar to the alpha2 than the alpha1 from vertebrates. The MEGA results are statistically significant as determined using both Wilcoxon rank and Kolmogorov-Smirnov tests (P equals 0.01 and 0.02, respectively). The distance to the arthropod fruit fly, however, was not significant, presumably due to the short branch leading to the fly (Additional file 3). Our series of three BLAST analyses identified only a single alpha subfamily homolog in each of the non-vertebrates studied here. Thus all alpha subfamily homologs in non-vertebrates should be alpha2. As such, the annotated alpha1 in *Pediculus humanus corporis *(body louse) would be incorrectly annotated. We propose that members of the alpha subfamily in non-vertebrates be annotated as alpha2 (Additional file 4).

### Distant relatives of catenins in non-metazoans

The p120 subfamily members have been detected in all metazoans, but not in the premetazoan *M. brevicollis *or other non-metazoans. However, relatives of this subfamily have been detected in non-metazoans by our BLAST searches. These included armadillo repeat-containing protein (armc) 3, armc4, the importin alpha family, and vac8p.

The beta subfamily members have been detected in all metazoans, but not in the premetazoan *M. brevicollis *or other non-metazoans. However, several potential beta catenin homologs and beta catenin-like genes have been detected in non-metazoans by BLAST. These genes included aardvark, vac8p, arabidillo-1/-2, physcodillo-1/-2, and beta-catenin-like.

We did extensive analyses by BLASTP and Bayesian phylogenetics to determine the relationship of distant homologs of both the p120 and beta subfamilies. Our analyses show that arabidillo-1/-2 and physcodillo-1/-2 formed a well-supported clade (PP = 1.0), and aardvark, vac8p, beta-catenin-like, armc3, armc4 and importin alpha all form individual monophyletic clades (PP = 1.0, phylogeny not shown). Thus, none of these distant homologs grouped with the beta or p120 subfamilies in our phylogenetic analyses.

Alpha subfamily members have been detected in all metazoans but not in the premetazoan *M. brevicollis*. However, a relative of this subfamily (vinculin-like) has been detected in the premetazoan *M. brevicollis*. An additional relative (alpha-catulin) was detected by our BLAST analyses but present only in metazoans. Bayesian phylogenetic analyses showed that alpha catenins, vinculin and alpha-catulin all formed three monophylogenetic clades (PP equals 0.99, 0.97 and 1.0, respectively).

## Discussion

### Origins and evolution for the catenin family

Despite similar names, alpha catenins are not homologous to p120 and beta catenins. This is evident from sequence and structural comparisons (Figure 2). Conversely, this figure shows that beta and p120 share a common ancestor as a result of a duplication event prior to the diversification of metazoans. This evolutionary relationship from sequence-based analyses is consistent with the differences in secondary structures between alphas versus p120s/betas. The p120 subfamily contains 9 ARM repeats [12] and the beta subfamily contains 12 of these ARM repeats [3]. The alpha subfamily, however, contains three vinculin homolog domains instead of ARM repeats, and belongs to the vinculin superfamily [4]. Therefore, catenin should not be called a family, since it is just a group of proteins binding C-terminal of classical cadherins. For the p120 subfamily, we conclude that it is more appropriate to refer to the p120 subfamily as delta. We suspect that this change in nomenclature will be difficult to accept at first (as all name changes are) but our proposed change more accurately reflects the evolution of this subfamily and the new nomenclature is more functionally consistent for experimental biologists.

Delta2 or ARVCF is the oldest member of the p120 subfamily according to our analyses but we did not detect it in the premetazoan *M. brevicollis*. This suggests a metazoan origin for the p120 subfamily. Unlike the p120 subfamily, we have detected the relatives of this subfamily beyond metazoans, such as armc3, armc4, the importin alpha family, and vac8p. This suggests that these armadillo repeat proteins share a common ancestor. This can be further inferred by their sequence and structural conservations though they have diverse functions in different kingdoms including animals, fungi and plants [13].

Beta catenin is the oldest member of the beta subfamily according to our analyses but we did not detect it in the premetazoan *M. brevicollis*. This indicates a metazoan origin for the beta subfamily. Previous studies also suggested the potential metazoan origin of beta catenin and its involvement in cell adhesion for multicellular organisms [14]. Several types of genes in non-metazoans, however, have been considered potential beta catenin homologs [13] and thus some have been annotated as beta catenin or beta catenin-like. These genes included aardvark in amoeba, vac8p in fungi and plants, arabidillo-1/-2 and physcodillo-1/-2 in plants, and beta-catenin-like in both animals and plants. The aardvark gene in amoeba participates in adherens junctions and cell signaling, and it may be the amoeba's version of a precursor to beta catenin [15]. Vac8p has 11 ARM repeats and this is close to the 12 ARMs that compose beta catenin [16]. None of these potential beta homologs grouped with the beta subfamily in our phylogenetic analyses, thus supporting our hypothesis of a metazoan origin for the beta subfamily.

Our analyses also revealed a significantly higher mutation rate within the Nematode clade for beta catenin paralogs specific to this clade. The high rate can be correlated to experimental work showing subfunctionalization of cellular adhesion and transcriptional activation (signalling) among the three paralogs in *C. elegans *revealed by their protein-binding partners [17]. It remains to be determined whether this is due to relaxation of selective constraints or due to positive selection.

Alpha2 catenin is the oldest member of the alpha subfamily according to our analyses but we did not detect it in the premetazoan *M. brevicollis*. This suggests a metazoan origin for the alpha subfamily. We did however detect a vinculin-like gene in the premetazoan *M. brevicollis*. This suggests that the alpha subfamily duplicated from vinculin-like genes after the separation of metazoans/premetazoans but prior to the diversification of metazoans.

In summary, our analyses did not identify any p120, beta or alpha subfamily members in the premetazoan *M. brevicollis*. We confirmed this absence by extending our analyses to other non-metazoans such as the amoeba *D. discoideum*, the protist *C. parvum*, three fungi (*Aspergillus, Saccharomyces*, and *Schizosaccharomycetes*), three unicellular algae (*Ostreococcus, Chlamydomonas*, and *Thalassiosira*), and the plant *Arabidopsis*. Again, none of these non-metazoans contained a member of the catenin family. We conclude that the catenin family arose during the origin of metazoans while some other armadillo repeat proteins and vinculin-like proteins were present prior to the evolution of metazoans.

### Evolution-related physiology of the catenin family

The members of the three catenin subfamilies emerged at different points during metazoan evolution. It would be interesting to correlate gene evolution of catenins to the cellular physiology associated with the functional divergence of this gene family.

Our results suggest that either delta2 or ARVCF is the oldest member of the p120 subfamily - one evolved with the origins of metazoan while the other evolved with the origins of chordates. Pkp4, p120, pkp1, pkp2, and pkp3 show vertebrate origins and this is consistent with the conclusions of other analyses [18]. Functional divergence between delta2 and ARVCF in chordates is highlighted by delta2's neuron-specific expression while ARVCF is ubiquitously expressed [2]. Both ARVCF and p120 are ubiquitously expressed in epithelial tissue [2], but their expression is mutually exclusive [19] and have complementary distributions in epithelial adherens junctions as interpreted from BioGPS [20]. Our evolutionary studies show that p120 duplicated from ARVCF during the origin of vertebrates. The origin of p120 members appears to coincide with the vertebrate origin of p120's binding partner Kaiso [18], a nuclear factor participating in signaling pathways [21]. This interaction might relate to epigenetic transcriptional regulation and Wnt signaling modulation via methyl-CpG islands [22] and the transcription factor TCF [23], participating in vertebrate-specific development and transcriptional regulation [18]. The nuclear signaling functions of ARVCF have not yet been identified but it is known that it interacts with the novel protein Kazrin localized in both the cytoplasm and nucleus [5]. P120 is unique among the members of the p120 subfamily because it interacts in the nucleus with the nuclear transcription repressor Glis2 [24]. This demonstrates that p120 has diverged from ARVCF due to its unique interaction with Glis2 and its vertebrate-specific interaction with Kaiso, but p120 still shares functional redundancy with ARVCF since they can rescue one another's null mutants [25]. Another unique feature of p120 is the absence of the C-terminal PDZ binding domain. The lack of this domain may enable p120 and its binding cadherins to evolve with more flexibility in a PDZ-independent manner [18]. In support of this view is the observation that the function of delta2 catenin in spine density regulation is independent of cadherins but dependent on an interaction with PDZ-domain containing proteins while p120 regulates spine density using an alternative mechanism that depends on Rho GTPases [26].

Both p120 and ARVCF, but neither delta2 nor pkp4, have different isoforms that result from alternative splicing at the 5' portion of the transcript [19,27]. Translating the conserved N-terminal coiled coil domain during such isoform switching, and in coordination with the dynamic cadherin switching (e.g., E-cadherin to N-cadherin), occurs during the transformation from epithelial and other sessile cell types to mesenchymal (e.g., fibroblasts) and other motile cell types (e.g., neurons) [2]. This process might be important in development, wound healing and cancer [18].

Pkp4 duplicated from delta2 during the origin of vertebrates and both have neuron-specific expression patterns, however this pattern of expression is reciprocal according to BioGPS. Delta2 is predominantly expressed in the brain while pkp4 is expressed in the brain but also ubiquitously expressed in other tissues. Pkp4 differs from delta2 in that pkp4 participates in desmosome formation in addition to adherens junctions according to numerous experimental reports [28], however one study failed to verify this dual functionality [29]. Pkp1, pkp2, and pkp3 are vertebrate-specific and are well known for their functions in desmosomes instead of adherens junctions [30]. The origins of these plakophilins appear to coincide with our assumption of the origin of desmosomes in vertebrates since our analyses reveal that other desmosome-specific genes are found only in vertebrates (further discussion is provided below).

Our results suggest that beta is the oldest member of the beta subfamily since it is present in all metazoans while gamma is a more recent acquisition since it is found only in vertebrates. Both beta and gamma are ubiquitously expressed at basal levels but gamma is uniquely highly-expressed in some tissues such as tongue. Beta and gamma both function in the formation of adherens junctions however gamma has functionally diverged from beta since gamma can also participate in desmosome formation [6].

Gamma (also called plakoglobin) and the four plakophilins (discussed above) are all components of the desmosome [6] and all have vertebrate origins according to our analyses. This suggests to us that the desmosome evolved in conjunction with the origin of vertebrates. To further validate this hypothesis we performed computational analyses of other non-catenin gene families involved in desmosome formation. These included both desmosomal cadherins desmogleins and desmocollins, as well as desmoplakins and thus we confirmed that the presence of genes involved in desmosome formation is limited to vertebrates and not observed in non-vertebrates. All of our results are consistent with the observation that desmosome-containing tissues (e.g., certain cardiac and skeletal muscles [31], keratin-containing hair [32], and others) have evolved with or after the origin of vertebrates.

Our results suggest that alpha2 is the oldest member of the alpha subfamily since it is present in all metazoans while alpha1 is a more recent acquisition since it is found only in vertebrates. Alpha1 and alpha2 display both similar and reciprocal expression and distribution patterns in the dorsal root ganglia and spinal cord at the lumbar level where sciatic nerves originate [33]. The two are similar in that they both display brain-specific expression patterns however they display reciprocal expression patterns in terms of the specific types of tissues in the brain [34]. Unlike alpha1, alpha2 functions by binding the nuclear transcription repressor ZASC1 [35]. Alpha2 knockouts in mice mainly affect the nervous systems by causing cerebellar deficient folia [4]. Alpha1 knockouts in mice are lethal at the blastocyst stage but its knockout mainly affects the epithelial tissues such as skin [4].

Alpha3 is the most recently evolved member of the alpha subfamily since it is found only in amniotes. The observation that alpha3 and alpha2 share identical exon-exon boundaries [36] suggests to us that alpha3 evolved via a gene duplication of alpha2 coinciding with the origin of amniotes. Unlike alpha1 and alpha2, alpha3 interacts with pkp2 in the area composita (a hybrid adherens junction in the heart muscle), yet co-expresses with alpha1 at intercalated discs of cardiomyocytes [37]. We could not find a physiological link between the amniote-specific alpha3 and the observation that it is highly expressed in the testis and participates in adherens junctions between sertoli and germ cells, highly expressed in the testis interstitial tissue or highly expressed in peritubular myoid cells of the testis [38] because the above cells or tissues are also found in the non-amniote fish. However, alpha3 interacts with 1-adadin and this interaction, along with a truncated isoform of alpha3 present in the testis [39], may be unique to spermatogenesis in amniotes. No alpha3 knockout experiments have been performed in mice [4].

Overall, delta2, alpha2 and N-cadherin are present in nearly all metazoans and all are expressed in neural-specific tissues. Their vertebrate-counterparts p120, alpha1 and E-cadherin, however, are mainly expressed in epithelial tissues in addition to neural tissues. Extensive co-evolution exists between the catenin family and their binding partners; these include p120 with Kaiso, delta2/alpha2 with N-cadherin, p120/alpha1 with E-cadherin, and pkps/plakoglobin (gamma catenin) with desmosomal cadherins.

## Conclusions

The p120, beta and alpha subfamilies are present in all metazoans, but none of the subfamilies are present in non-metazoans. This indicates a metazoan origin for the catenin family. Each catenin subfamily has undergone duplications at different levels (species-specific, subphylum-specific or phylum-specific) and to different extents (in the case of the number of homologs).

Vertebrate-specific duplications occurred in all three subfamilies: p120, pkp4, pkp1, pkp2, and pkp3 in the p120 subfamily; gamma in the beta subfamily; and alpha1 in the alpha subfamily. All three subfamilies had extensive annotation issues and these have been effectively resolved by our studies. We anticipate that this resolution in combination with our evolutionary analyses will make it easier for experimental biologists to correlate diverse catenin physiologies to interesting evolutionary innovations such as the development of multicellularity and the role of adhesion during this development and the evolution of terrestrial organisms and their ability to prevent desiccation.

## Methods

### Gene presence/absence for the catenin family

A list of species with complete genome sequences was chosen to represent the species tree for metazoan evolution together with the premetazoan unicellular choanoflagellate *M. brevicollis *serving as the outgroup (Figure 1). The presence/absence of catenin was determined in all the selected species by searching gene names in public databases and/or via species-specific BLAST searches. For all BLAST analyses conducted in the present study, hits having E-values less than 0.05 and sequence identity greater than 15% were considered significant. Detailed BLAST searches against genomic databases were performed for non-vertebrates only in order to determine the history of catenins before the origin of vertebrates. Species-specific duplications in vertebrates have occurred for catenin subfamilies but are not the focus of our current study [18].

### Datasets

The protein sequences were extracted for evolutionary analyses and sequence identifiers can be found in the additional files. All sequence identifiers are NCBI protein sequence GI numbers with only two exceptions. The first exception is the pkp1 gene from frog. It was downloaded from the Joint Genome Institute (JGI) *Xenopus tropicalis *assembly v4.1 [40], and it can be queried using the protein ID '156321'. The second exception is the gamma catenin from finch. The sequence is derived from mRNA sequence in the NCBI nucleotide database (nucleotide identifier: 224086507) and translated by the Translate tool on the ExPASy server [41].

### Phylogenetic analyses and tertiary structures

Sequence alignments were performed by ClustalW [42]. Phylogenetic analysis was carried out using MrBayes 3.1.2 Unix version [43]. The MPI version of MrBayes was run in parallel on eight nodes with MPICH2 installed [44]. Bayesian trees with posterior probabilities were constructed with mixed amino acid models, a gamma distribution for rate variation among sites, and a proportion of invariable sites. MrBayes was executed with two runs (four chains for each run), one million generations of Markov Chain Monte Carlo (MCMC) analyses, with 1000 as the sample frequency. The number of MCMC generations indicated convergence of the two runs by having a standard deviation of split frequencies less than 0.05. The posterior probability of each split was estimated by sumt with 250 trees discarded as burnin based on the plot of 'generation vs. log probability'. Parameters were summarized by sump with 250 burnin, and values for the Potential Scale Reduction Factor (PSRF) were all close to 1.0 for all parameters. Bayesian phylogenies with branch lengths and posterior probabilities are shown in Additional files 1, 2, 3, with each tree having sea anemone as the outgroup. Sequence/taxon names in the trees are a combination of gene name, species name, and NCBI sequence identifier. Similar color patterns were applied for species of different phyla as in Figure 1. For all phylogenies, scale bars represent amino acid replacements per site per unit evolutionary time. The tertiary structures for representative genes were generated by using PyMOL [45] (Figure 2).

### Annotation validation

Sequence similarity comparisons were conducted by BLAST [46]. Pairwise distance comparisons were conducted using MEGA4 [47] with the Dayhoff model [48] and the JTT model [49]. Statistical significance in the differences between two groups of pairwise distances was evaluated by Wilcoxon rank and Kolmogorov-Smirnov tests implemented in R [50]. Bayesian trees with posterior probabilities were constructed to determine evolutionary relationships. Functional divergence among homologs was inferred using the DIVERGE software package [51].

### Tissue specific gene expression

We accessed the BioGPS database to identify divergent patterns in gene expression and attempt to correlate potential functional differences between catenin members. BioGPS (an online resource containing gene expression data based on Affymetrix microarrays) was used to visualize and compare gene expression patterns for catenin family members and catenin-related genes in human and mouse cells [20].

## Abbreviations

P120: p120 catenin; beta: beta catenin; alpha: alpha catenin; ARVCF: Armadillo Repeat protein deleted in Velo-Cardio-Facial syndrome; pkp: plakophilin; aa: amino acids; PP: Posterior Probability; ARM: armadillo; MCMC: Markov chain Monte Carlo; PSRF: Potential Scale Reduction Factor.

## Authors' contributions

ZMZ, EAG and ABR conceived the study, ZMZ and EAG carried out the analyses, and ZMZ, EAG, and ABR wrote the manuscript. All authors read and approved the final manuscript.

## Supplementary Material

Additional file 1 **The Bayesian phylogeny for the p120 subfamily**. Click here for file

Additional file 2 **The Bayesian phylogeny for the beta catenin subfamily**. Click here for file

Additional file 3 **The Bayesian phylogeny for the alpha catenin subfamily**. Click here for file

Additional file 4 **Summary of annotation problems for the catenin family**. 'armp' stands for 'armadillo segment polarity protein'. *Multiple GI numbers in the same box referred to the case that the same gene has been sequenced multiple times by different groups, and they are about 99-100% identical. Click here for file
