# Supplementary figures and images for: The evolutionary history of the catenin gene family during metazoan evolution
Source: BMC Evol Biol. 2011 Jul 8;11:198. doi: 10.1186/1471-2148-11-198 (PMC3141441; doi:10.1186/1471-2148-11-198)

Additional file 1: The Bayesian phylogeny for the p120 subfamily.

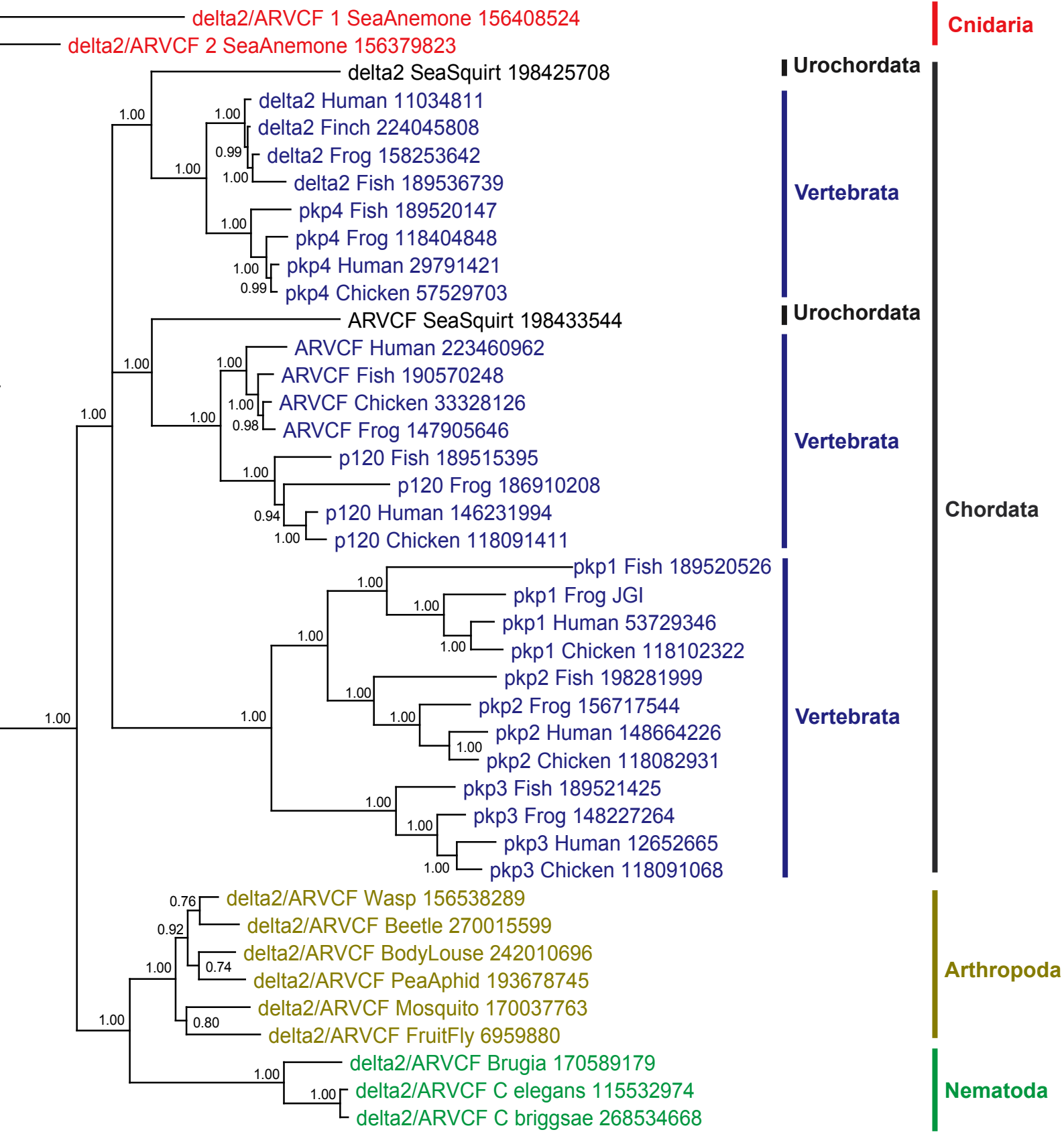

Supplement: Additional file 1 — The Bayesian phylogeny for the p120 subfamily. [file 1471-2148-11-198-S1.PDF]

Additional file 2: The Bayesian phylogeny for the beta catenin subfamily.

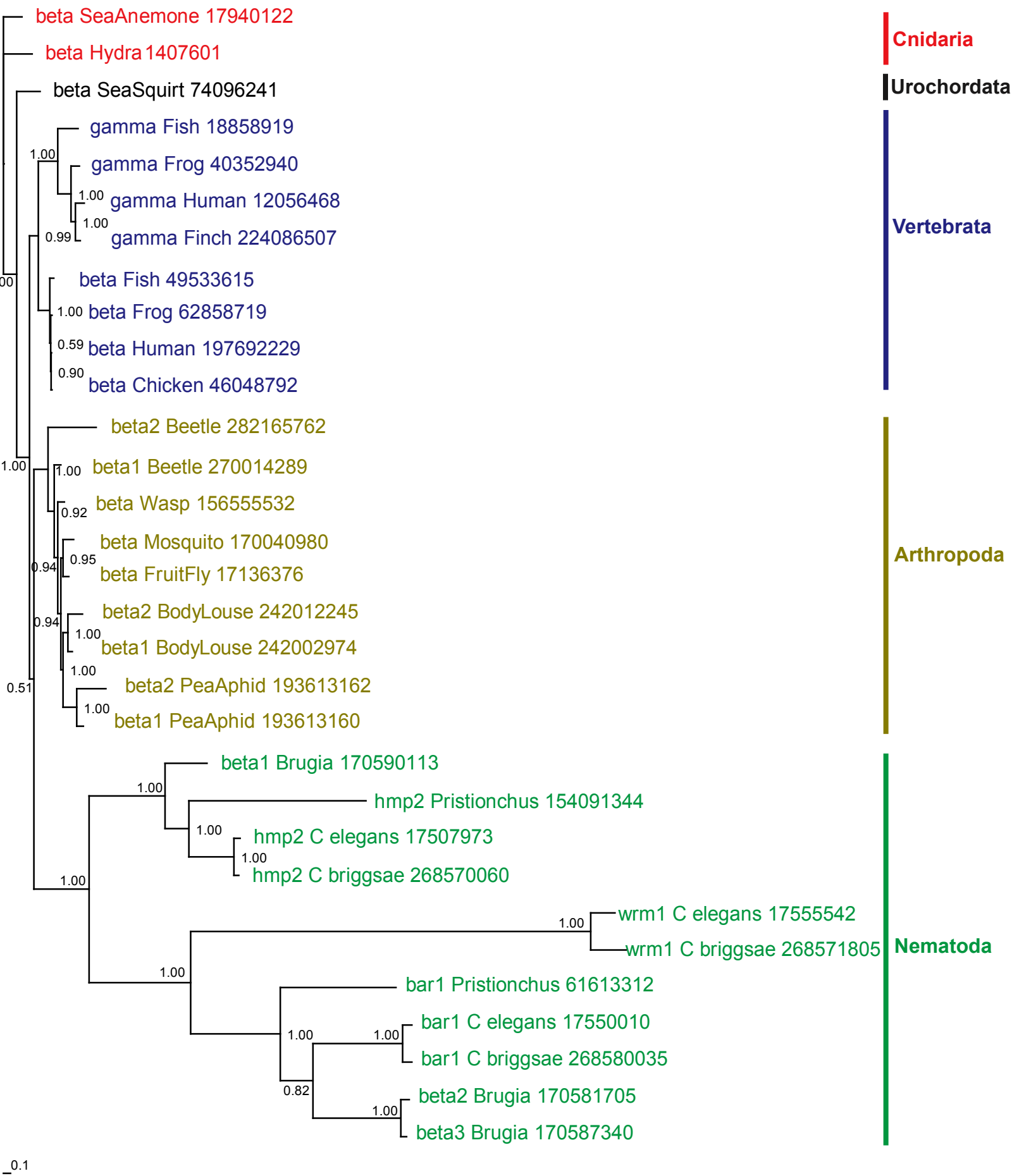

Supplement: Additional file 2 — The Bayesian phylogeny for the beta catenin subfamily. [file 1471-2148-11-198-S2.PDF]

Additional file 3: The Bayesian phylogeny for the alpha catenin subfamily.

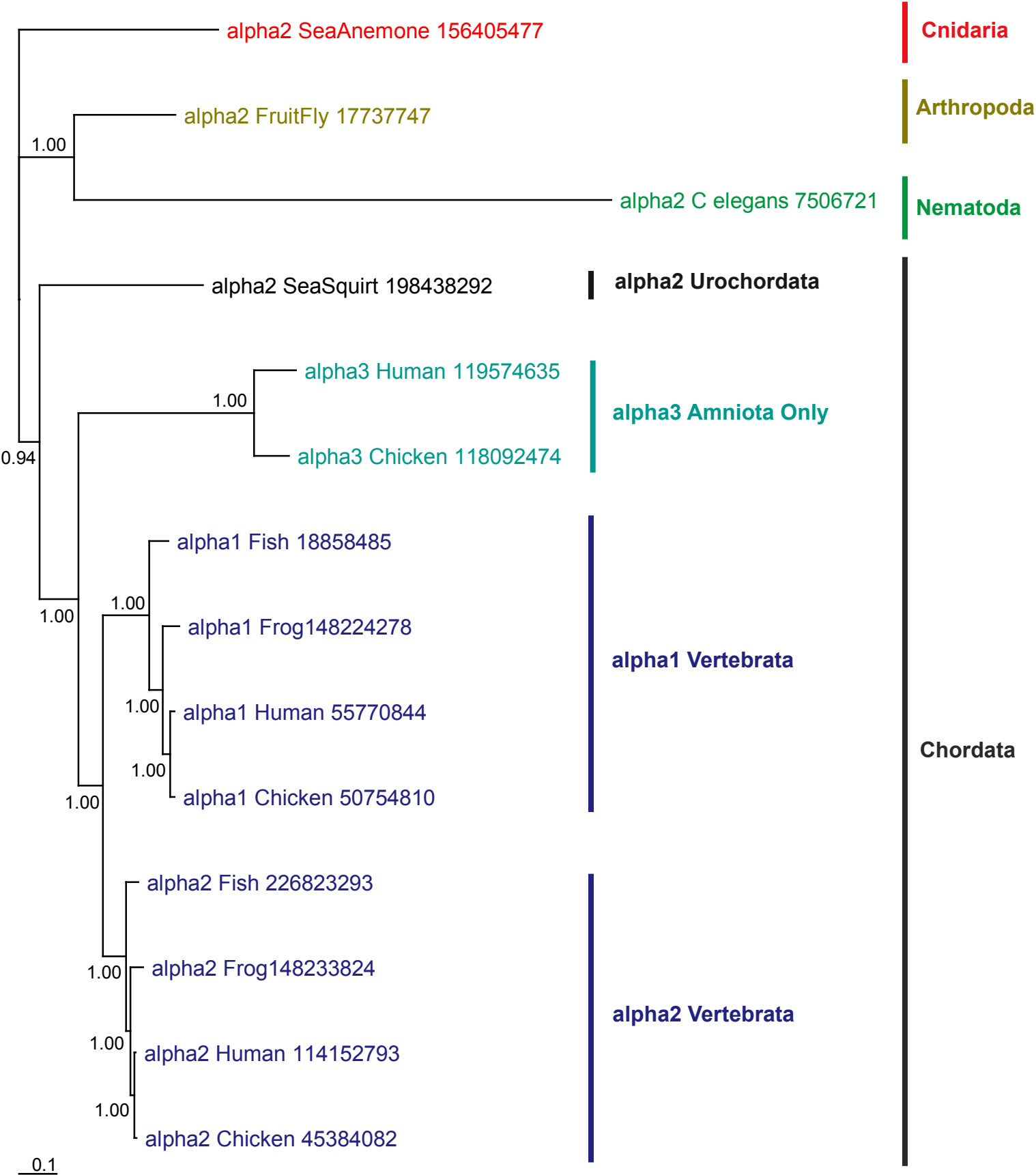

Supplement: Additional file 3 — The Bayesian phylogeny for the alpha catenin subfamily. [file 1471-2148-11-198-S3.PDF]
