# Supplementary material for: The evolutionary history of the catenin gene family during metazoan evolution
Source: BMC Evol Biol. 2011 Jul 8;11:198. doi: 10.1186/1471-2148-11-198 (PMC3141441; doi:10.1186/1471-2148-11-198)
Supplement: Additional file 4 — Summary of annotation problems for the catenin family. 'armp' stands for 'armadillo segment polarity protein'. *Multiple GI numbers in the same box referred to the case that the same gene has been sequenced multiple times by different groups, and they are about 99-100% identical. [file 1471-2148-11-198-S4.PDF]

Additional file 4 – Summary of annotation problems for the catenin family.

Phylum	Species	Sequence identifiers (NCBI GI number)*	Original annotation (gene symbol, gene description or definition)	Revised annotation (gene)
Arthropoda	*Drosophila melanogaster*	116007493; 113194561; 116007494; 116007496; 30923507; 6959880	p120	delta2/ARVCF
	Culex quinquefasciatus	170037762	Pkp4	delta2/ARVCF
	Nasonia vitripennis	156538289	p120	delta2/ARVCF
	Tribolium castaneum	189241994	similar to pkp4	delta2/ARVCF
	Pediculus humanus corporis	242010696	armadillo repeat protein, putative	delta2/ARVCF
	Acyrthosiphon pisum	193678745	similar to Adherens junction protein p120 CG17484-PB	delta2/ARVCF
Nematoda	*Caenorhabditis elegans*	75025630; 115532974; 285307422; 115532976; 87251663	jac-1	delta2/ARVCF
	Caenorhabditis briggsae	268534668	Cbr-jac-1	delta2/ARVCF
	Brugia malayi	170589179	Fibronectin type III domain containing protein	delta2/ARVCF
Platyhelmint hes	*Schistosoma mansoni*	256078604	catenin and plakophilin	delta2/ARVCF
Cephalochor date	*Branchiostoma floridae*	260834528	hypothetical protein BRAFLDRAFT_146 863	delta2/ARVCF
		260834522	hypothetical protein BRAFLDRAFT_246 677	delta2/ARVCF
Chordata	*Xenopus laevis*	27447669	p120	pkp4
	Ciona intestinalis	198433544	similar to catenin, delta 1	ARVCF
	Danio rerio	190570248	armadillo repeat protein	ARVCF

Cnidaria	*Hydra magnipapillata*	221130487	similar to Adherens junction protein p120 CG17484-PB	delta2/ARVCF
		221131941	similar to predicted protein	delta2/ARVCF
	Nematostella vectensis	156379823	hypothetical protein	delta2/ARVCF
		156408524	hypothetical protein	delta2/ARVCF
Arthropoda	*Tribolium castaneum*	270014289	armadillo-1	beta catenin
		282165762	armadillo-2	beta catenin
	Pediculus humanus corporis	242012245	armp, putative	beta catenin
		242002974	armp, putative	beta catenin
	Acyrthosiphon pisum	193613160	similar to armadillo protein	beta catenin
		193613162	similar to armadillo protein	beta catenin
	Aedes aegypti	122106728	armp	beta catenin
	Gryllus bimaculatus	37991668	armadillo protein	beta catenin
	Drosophila melanogaster	17136376; 45551205	armp;	beta catenin
	Drosophila pseudoobscura pseudoobscura	221222436; 198467818; 198146121	armp; Dpse\GA27602	beta catenin
	Drosophila yakuba	194187865; 195477916	Dyak\GE16998	beta catenin
	Culex quinquefasciatus	170040980	armadillo	beta catenin
	Nasonia vitripennis	156555532	armp	beta catenin
	Apis mellifera	297515465	armp	beta catenin
Nematoda	*Brugia malayi*	170590113	Armadillo/beta-catenin-like repeat family protein	hmp2 (beta catenin)
		170581705	Armadillo/beta-catenin-like repeat family protein	bar1 (beta catenin)
		170587340	Armadillo/beta-catenin-like repeat family protein	bar1 (beta catenin)
Lophotrochozoa	*Schistosoma mansoni*	256074627	plakoglobin	beta catenin
Cnidaria	*Nematostella vectensis*	156615300	hypothetical protein	beta catenin
Fugus	*Cryptococcus neoformans var. neoformans JEC21*	58258547	beta catenin	vac8p

Arthropoda	*Drosophila melanogaster*	17737747; 15291871	alpha catenin	alpha2 catenin
	Drosophila ananassae	194767509	Dana\GF20570	alpha2 catenin
	Drosophila erecta	194876539	Dere\GG16297	alpha2 catenin
	Drosophila grimshawi	195038657	alpha catenin	alpha2 catenin
	Drosophila mojavensis	195107690	Dmoj\GI23967	alpha2 catenin
	Drosophila persimilis	195151767	Dper\GL21877	alpha2 catenin
	Drosophila yakuba	195496883	Dyak\GE19475	alpha2 catenin
	Drosophila virilis	195400267	Dvir\GJ11153	alpha2 catenin
	Drosophila willistoni	195445209	Dwil\GK11940	alpha2 catenin
	Pediculus humanus corporis	242018616; 212514782	alpha1 catenin, putative	alpha2 catenin
	Ixodes scapularis	241730211	alpha catenin, putative	alpha2 catenin
	Acyrthosiphon pisum	193673870	similar to alpha Catenin CG17947-PA	alpha2 catenin
	Apis mellifera	66525427	alpha catenin	alpha2 catenin
	Tribolium castaneum	91076138	similar to actin binding	alpha2 catenin
	Aedes aegypti	157138056; 108880700; 157138058; 108880701	actin binding	alpha2 catenin
	Anopheles gambiae str. PEST	158290113	AgaP_AGAP003424	alpha2 catenin
	Culex quinquefasciatus	170038843	actin binding protein	alpha2 catenin
Cephalochordata	*Branchiostoma floridae*	260826764	hypothetical protein	alpha2 catenin
Echinodermata	*Strongylocentrotus purpuratus*	115894474	similar to alpha catenin	alpha2 catenin
	Lytechinus variegatus	1098900	alpha catenin	alpha2 catenin
Hemichordata	*Saccoglossus kowalevskii*	268053957	alpha catenin	alpha2 catenin
Platyhelminthes	*Schistosoma mansoni*	256073504	alpha catenin	alpha2 catenin
Nematoda	*Caenorhabditis elegans*	193208515; 17563198; 74961297;	hmp-1; hypothetical protein R13H4.4;	alpha2 catenin

		2738780; 7506721; 6434310	C. elegans protein R13H4.4a	
	Caenorhabditis briggsae	268557136; 187021170	Cbr-hmp-1	alpha2 catenin
	Brugia malayi	170573974	Vinculin family protein	alpha2 catenin
Cnidaria	*Nematostella vectensis*	156405477	hypothetical protein	alpha2 catenin
